# Supplementary material for: Fusing multisensory signals across channels and time
Source: PLoS Comput Biol. 2025 Jun 6;21(6):e1013125. doi: 10.1371/journal.pcbi.1013125 (PMC12143570; doi:10.1371/journal.pcbi.1013125)
Supplement: S1 Appendix — (PDF) [file pcbi.1013125.s001.pdf]

FUSING MULTISENSORY SIGNALS ACROSS CHANNELS AND TIME

Swathi Anil

Dan F. M. Goodman

Marcus Ghosh

April 16, 2025

1 Supplementary information

1.1 Why does task performance decrease with burst length?

The reason why LF and NLF's performance decrease with burst length k is that we control the task parameters across k to keep the expected number of times that $E = 1$ (signal is on) constant with k (see calculations in section "Normalising for signal sparsity"), but this doesn't necessarily keep performance constant.

To illustrate this, consider the following thought experiment represented abstractly in Fig S1 (and code listing below). Imagine you gathered all the trials where $E = 1$ precisely n times, and measured the performance of the algorithms only on these trials. You would see a sigmoidal increasing curve (blue curve in the figure). Now compute the probability that on a given trial with $k = 1$, that $E = 1$ precisely n times, for each n, and plot that on the same axis (orange curve). Do the same for $k = 8$ (green curve). What you will see is that the probability curve for $k = 1$ will look Gaussian around some mean value (that we are fixing), but that for $k = 8$ it will be close to zero everywhere except for multiples of 8. Both will sum to 1 of course. Now the average accuracy of the method will be the weighted average of the blue curve with weights given by either the orange ($k = 1$) or green ($k = 8$) curves, shown on the figure by the dashed horizontal lines. As can be seen, with this concave accuracy curve, the probability is slightly higher for $k = 1$ than for $k = 8$ because it is picking up more of the slightly worse part of the accuracy curve when $k = 8$ than when $k = 1$.

The code to generate the Fig S1 is as follows:

```
from pylab import *
from scipy.stats import norm, binom
N = arange(1, 31)
p = 0.54
acc_given_N = 1-norm.cdf(0.5, p, p*(1-p)/N)
plot(N, acc_given_N, c='C0', label='accuracy if (E=1) n times during trial')
def weighting(k, mu=14, N_max=50):
  w = zeros(N.size)
  n = arange(1, N.size+1)
  w[k::k] = binom.pmf(n, N_max, mu/(k*N_max))[:w[k::k].size]
  return w/sum(w)
plot(N, weighting(1), c='C1', label='probability (E=1) n times if k=1')
step(N, weighting(8), c='C2', label='probability (E=1) n times if k=8')
mean_acc_1 = sum(acc_given_N*weighting(1))
mean_acc_8 = sum(acc_given_N*weighting(8))
print('k=1 mean =', mean_acc_1)
print('k=8 mean =', mean_acc_8)
axhline(mean_acc_1, ls='--', c='C1', label='mean accuracy k=1')
axhline(mean_acc_8, ls='--', c='C2', label='mean accuracy k=8')
legend(loc=(0.42, 0.5))
xlabel('n = number of times (E=1) during trial')
tight_layout();
```

1.2 Task code

The following python code demonstrates how we generate trials for the tasks described in this study. The complete working code is provided in the repository.

1.2.1 Detection Task

```python

def detection_task(n, pm, pe, pn, pc, pi):
    """
    Generate a single trial for the Detection task.

    Parameters:
    n: int - number of time steps
    pm: float - probability of target presence (M != 0)
    pe: float - probability of signal emission when target is present
    pn: float - probability of non-zero observation when no signal is present
    pc: float - probability of correct observation when signal is present
    pi: float - probability of incorrect observation when signal is present

    Returns:
    M: int - target motion direction (-1, 0, or 1)
    E: list - emission states for each time step
    A: list - observations in channel A for each time step
    V: list - observations in channel V for each time step
    """
    # Choose target motion direction
    M = choice([-1, 0, 1], p=[pm/2, 1-pm, pm/2])
    E = []; A = []; V = []

    for t in range(n):
        # Determine if a signal is emitted (depends on M)
        if M:
            e = choice([0, 1], p=[1-pe, pe])
        else:
            e = 0

        # Set distribution for A and V (depends on M and E)
        if e:
            # Signal is emitted
            vals = [-M, 0, M]  # Possible values
            p = [pi, 1-pc-pi, pc]  # Probabilities
        else:
            # No signal or no target
            vals = [-1, 0, 1]
            p = [pn/2, 1-pn, pn/2]

        # Generate observations for A and V
        A.append(choice(vals, p=p))
        V.append(choice(vals, p=p))
        E.append(e)

    return M, E, A, V
```

1.2.2 Time-dependent Detection Task

```python


def time_dependent_detection_task(nb_steps, pm, pe, pn, pc, pi, k):
    """
    Generate a single trial for the time-dependent Detection task (see  Det_k in
   Methods).
    """

```

```
    # Choose target motion direction
    M = choice([-1, 0, 1], p=[pm/2, 1-pm, pm/2])

    while True:
        # Generate base emission sequence
        base_e = choice([0, 1], size=nb_steps, p=[1-pe, pe])

        # Extend emissions for burst length k
        idx = np.where(base_e == 1)[0]
        fin_e = base_e.copy()
        for i in range(1, k):
            idx_plus = idx + i
            idx_plus = idx_plus[idx_plus < nb_steps]
            fin_e[idx_plus] = 1

        # Remove buffer and check if valid
        E = fin_e[k:].tolist()
        if sum(E) > 0:
            break

    # Generate observations
    A = []
    V = []
    for e in E:
        if e:
            vals = [-M, 0, M]
            p = [pi, 1-pc-pi, pc]
        else:
            vals = [-1, 0, 1]
            p = [pn/2, 1-pn, pn/2]

        A.append(choice(vals, p=p))
        V.append(choice(vals, p=p))

    return M, A, V, E
```

1.2.3 Lévy flights

Generation of Lévy flights can be broken down into few simple steps.

First, we define a Levy distribution to determine the lengths of emission bursts.

```
def levy_dist(lmax):
    l = np.arange(lmax + 1)
    pl = np.zeros(lmax + 1)
    pl[1:] = 1.0 / l[1:]**2
    pl /= np.sum(pl)
    return pl
```

Next, we create a sparse base emission sequence using a Bernoulli process.

```
pe_sparse = emission_probability
N = total_sequence_length + lmax - 1
num_nonzero = np.random.binomial(N, pe_sparse)
E_starts = np.random.choice(np.arange(N), size=num_nonzero, replace=False)
```

We then apply the Levy flight principle to extend each emission point according to lengths drawn from the Levy distribution.

```
L = np.random.choice(np.arange(lmax + 1), size=num_nonzero, p=levy_dist(lmax))
E = np.zeros(N, dtype=bool)
for e_start, l in zip(E_starts, L):
    E[e_start:e_start+l] = 1
E = E[lmax-1:]
```

Finally, we generate observations based on this emission sequence, taking into account the target motion and probabilities for correct, incorrect, and neutral observations.

```
def generate_obs(E, M, pc, pi, pn):
    return [np.random.choice([-M, 0, M], p=[pi, 1-pc-pi, pc]) if e else
            np.random.choice([-1, 0, 1], p=[pn/2, 1-pn, pn/2]) for e in E]
```
