# Supplementary figures and images for: Fusing multisensory signals across channels and time
Source: PLoS Comput Biol. 2025 Jun 6;21(6):e1013125. doi: 10.1371/journal.pcbi.1013125 (PMC12143570; doi:10.1371/journal.pcbi.1013125)

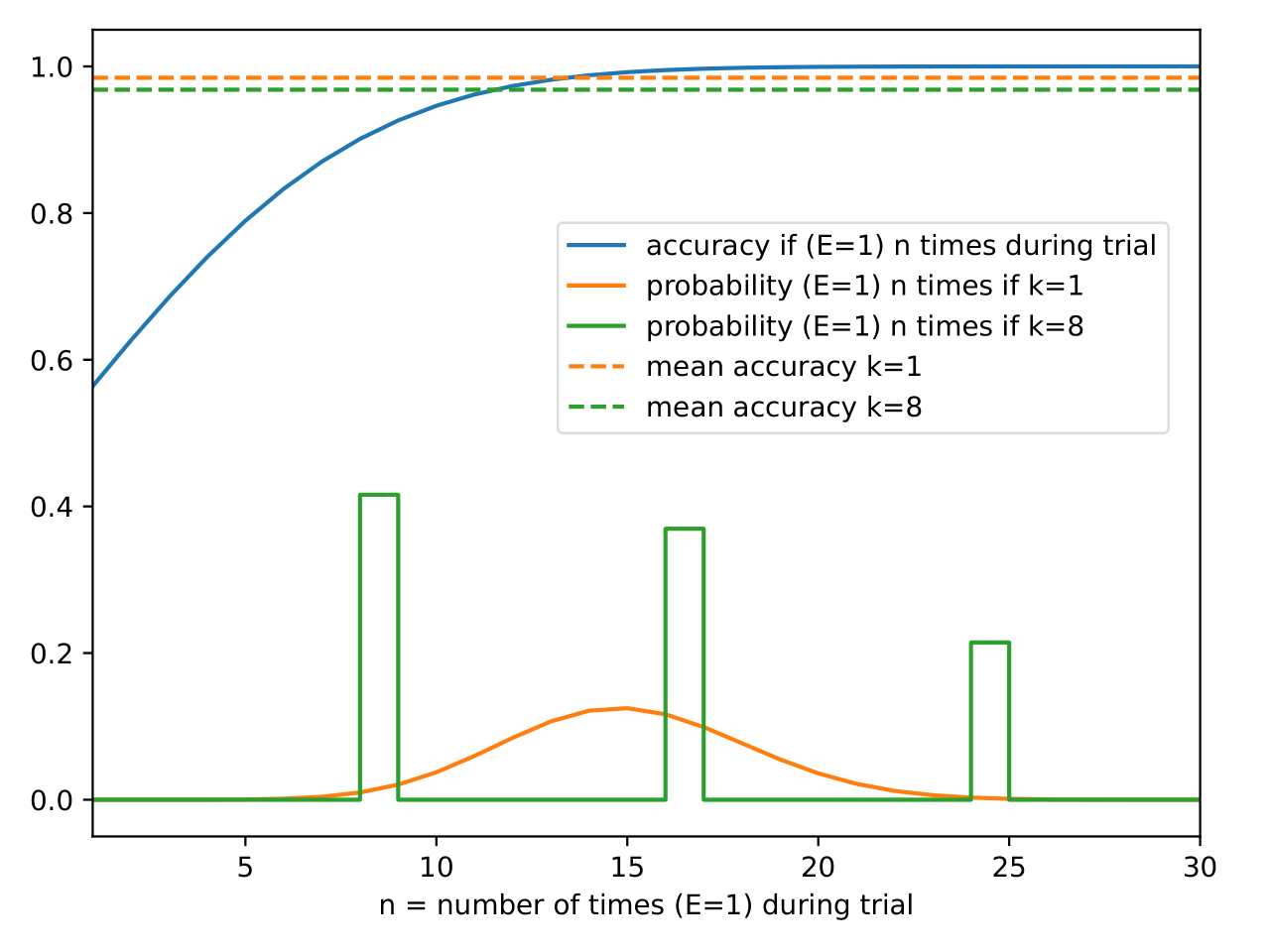

Supplement: S1 Fig — Explanation of why accuracy decreases for methods that do not use temporal information (LF, NLF) as k increases. See S1 Appendix. (TIFF) [file pcbi.1013125.s002.tiff]

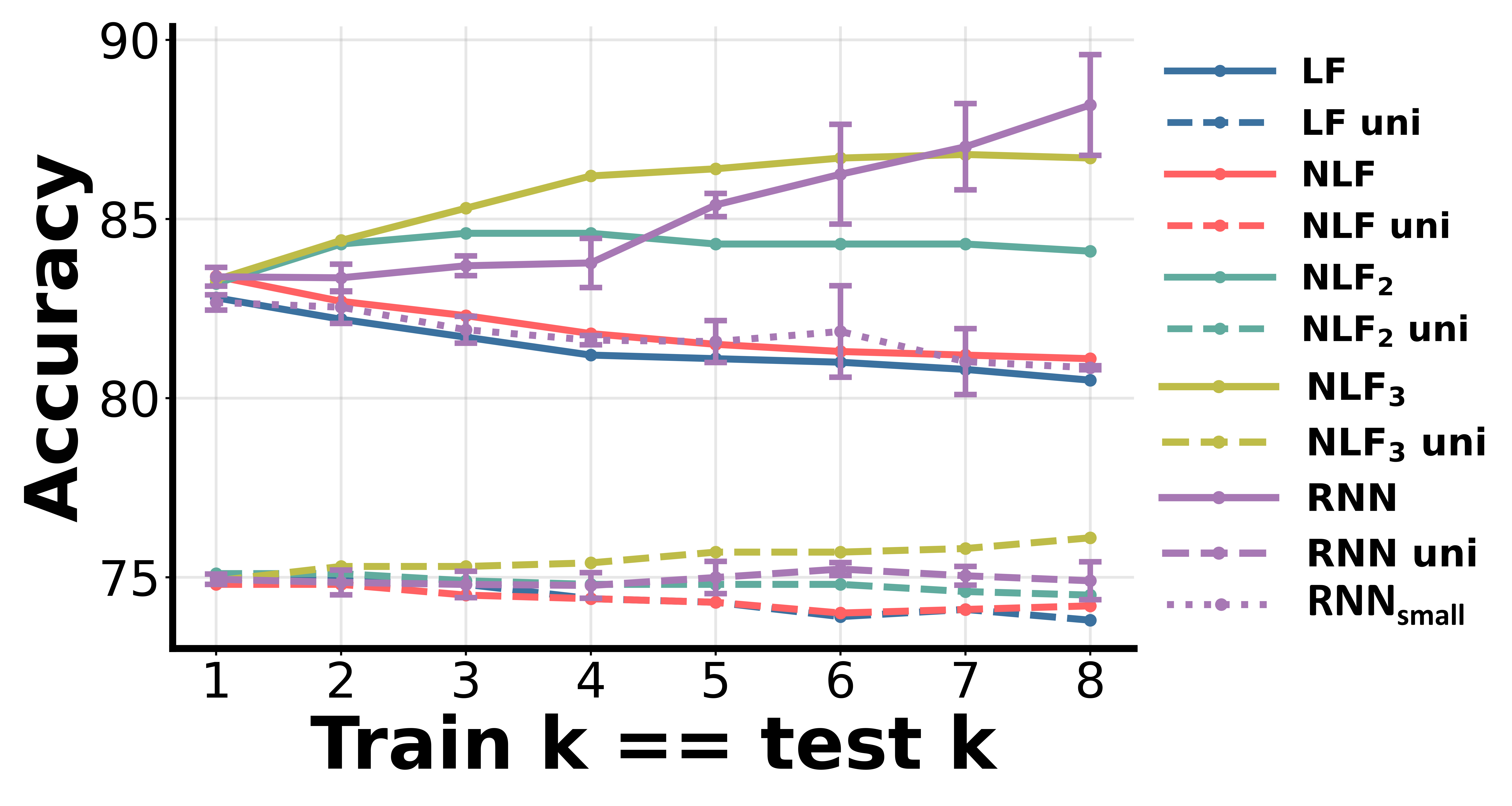

Supplement: S2 Fig — The accuracy of each model (y-axis) when trained and tested on the same value of the signal burst length k (x-axis); i.e. when tested in distribution. For the RNN models error bars show standard deviation over 5 separate train/test runs. For comparison, we include the performance of a scaled down RNN (RNNsmall), with 22 hidden units and 663 trainable parameters—a comparable number of parameters to NLF3 (729 parameters). (TIFF) [file pcbi.1013125.s003.tiff]

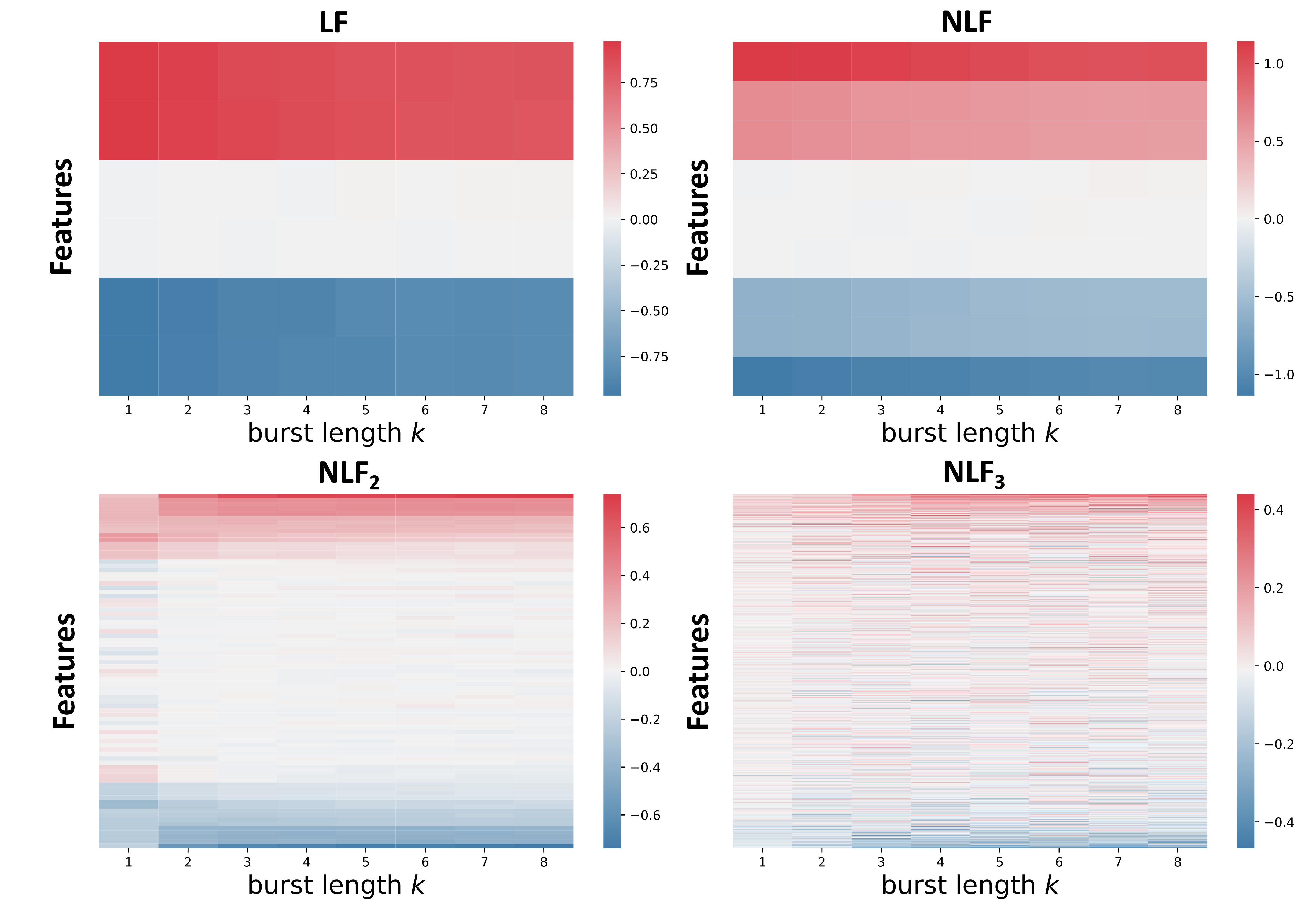

Supplement: S3 Fig — Learned parameter weights. Each subplot shows the weights learned by a single model (LF, NLF, NLF2 and NLF3). Each x-axis shows the value of k used to train the model. Each y-axis shows possible observations / features. For example (LRRR); sorted by their total corresponding parameter value (summed across values of k). Intuitively, ambiguous signals tend to be assigned low/zero weights. For example, (LRRL). While, unambigous signals, tend to be assigned larger weights. For example, (LLLL). And NLFw seems to learn different patterns when k<w vs when k≥w. Though we did not quantify these observations. (TIFF) [file pcbi.1013125.s004.tiff]

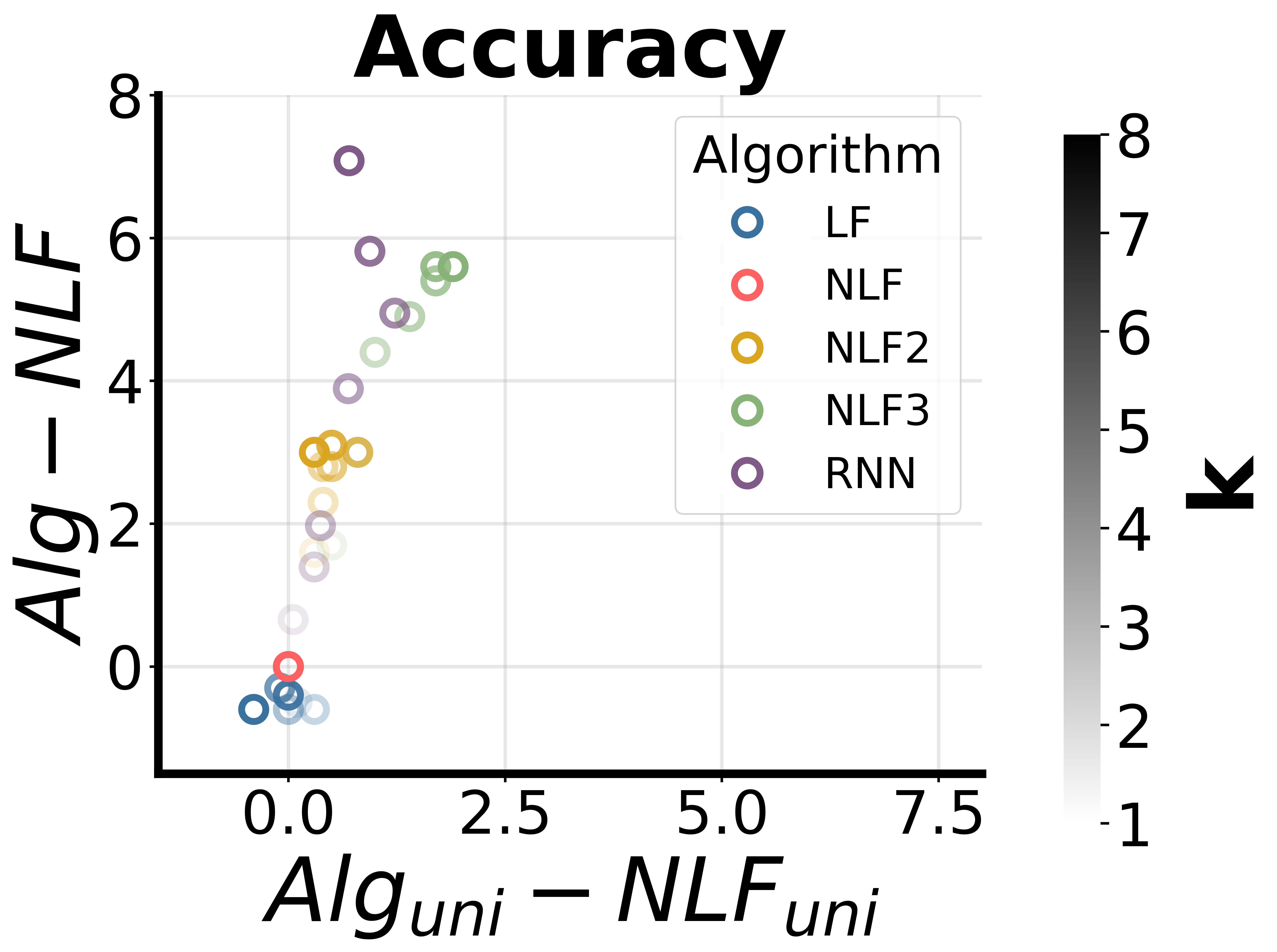

Supplement: S4 Fig — Scatter plot showing the performance of algorithms in unisensory (x axis) and multisensory (y axis) tasks. Accuracies are shown relative to the accuracy of the corresponding performance of the nonlinear fusion (NLF) algorithm. Lighter colours correspond to lower values of the burst length k, and darker values to higher values. (TIFF) [file pcbi.1013125.s005.tiff]
