# Supplementary material for: Fusing multisensory signals across channels and time
Source: PLoS Comput Biol. 2025 Jun 6;21(6):e1013125. doi: 10.1371/journal.pcbi.1013125 (PMC12143570; doi:10.1371/journal.pcbi.1013125)
Supplement: Attachment — Submitted filename: Anil_response_to_reviewers.pdf [file pcbi.1013125.s006.pdf]

Dear reviewers,

Thank you for your constructive feedback. In line with your suggestions, we have now included a set of unisensory models, have updated several figures and made changes throughout the text; all of which have improved our manuscript.

Below, we provide a comment-by-comment reply (in green) which details how we approached each of your suggestions.

Best,
The authors

Reviewer #1:
The authors describe an algorithm that combines information across "channels" (i.e., different modalities) and time to solve a new multisensory discrimination task. This work builds on their previously published task and algorithm (Ghosh et al., 2024), which focused on nonlinear fusion across channels only. In this new task, bits of sensory evidence are not temporally independent anymore but occur in bursts, and algorithms that treat each timestep independently perform sub-optimally. The authors propose here to nonlinearly fuse signals over short periods of time, and show that this modified model performs just as well as RNNs, with much less parameters.

By making their task temporally rich and thus more realistic, the authors provide an interesting framework to test the performance of various models. However, I found the current logic of the paper difficult to follow, especially as a biologist, and unless there are misunderstandings on my side the conceptual improvements over their previous work could be better described. Overall, it would really help if the authors could provide more detailed explanations and figures, and comparisons with the previous literature.

Major comments
- I found the current framing of the problem in the context of multisensory integration quite misleading. As far as I understood, the multisensory aspect of the task does not seem to play a role in the results described. Couldn't the paper be written with a single sensory channel and a temporal window? To clarify this point (both in the overall scope, the results and their explanation), it would be helpful to start with only one modality and put the emphasis on the effect of temporal integration only. Multichannel integration could then be studied in a later figure if needed.

As suggested, we have tested unisensory versions of our models (with and without temporal integration). These unisensory models perform worse than their multisensory counterparts (Figure 2A). And, interestingly, they benefit less from fusing information over time than their multisensory counterparts. In Figure 2A, for example, at k=8, the difference between NLF and NLF3 is far greater in the multisensory, than the unisensory case. We agree that these models are helpful to include and so have updated Figure

2A and added a supplementary figure showing the uni vs multisensory benefit for each model (supporting information 1.4).

Also, isn't the new model equivalent to the previous one but with channels 2, 3, etc. being a delayed version of the first channel? I would find it helpful if the authors could clarify this point from the beginning.

This is true for NLF2 and 3 (which fuse multisensory signals over short temporal windows). To clarify this, we have added the following sentence to the first paragraph in "Incorporating time into multisensory algorithms":

"As such, at each time step (*t*), these models combine incoming (*t*) and prior signals (from *t-w*)."

- It would also be very useful to provide more information and explanation in the figures and main text. Without the previous paper at hand, it is very difficult to follow what is the task, and what are the models, and makes it challenging to build an intuition of how things work. The figures directly jump from a schematic description of the model to a summary of their accuracy. Additional panels would help. In particular, showing example trials with the actual signals being emitted, and the behavior of the models over the course of the trial, would help clarify how the different models integrate the sensory evidence over time. This could for example help understand why LF and NLF's performance decreases with k (which I think I get but not sure).

As suggested, we have added a panel which, for an example trial, shows the emission variable (E), each channel's signals and the behavior of one model over the course this trial (Figure 1G). We have also added additional text to explain the task more clearly to the start of the "Multisensory algorithms are blind to temporal structure" section.

The reason why LF and NLF's performance decrease with k is that we control (task) parameters across k to keep the expected number of times that E=1 (signal is on) constant with k (see calculations in section "Normalising for signal sparsity"), but this doesn't necessarily keep performance constant. You can see why with a thought experiment. Imagine you gathered all the trials where E=1 precisely n times, and measured the performance of the algorithms only on these trials. You would see a sigmoidal increasing curve (blue curve on the figure below). Now compute the probability that on a given trial with k=1, that (E=1) precisely n times, for each n, and plot that on the same axis (orange curve). Do the same for k=8 (green curve). What you will see is that the probability curve for k=1 will look Gaussian around some mean value (that we are fixing), but that for k=8 it will be close to zero everywhere except for multiples of 8. Both will sum to 1 of course. Now the average accuracy of the method will be the weighted average of the blue curve with weights given by either the orange (k=1) or green (k=8) curves, shown on the figure by the dashed horizontal lines. As can be seen, with this concave accuracy curve, the probability is slightly higher for k=1 than for k=8 because it is picking up more of the slightly worse part of the accuracy curve when k=8 than when k=1.

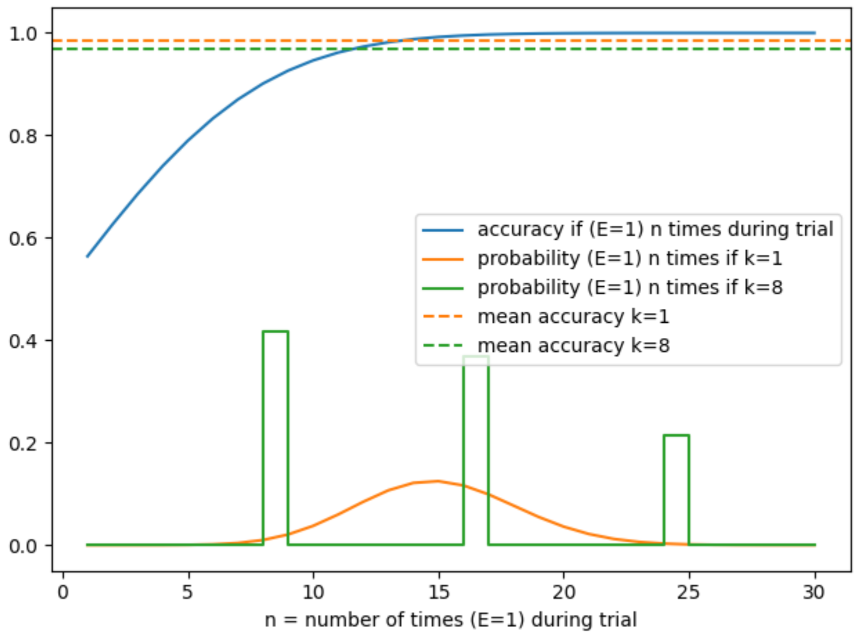

Note that this figure does not represent one of the methods here, it is just an abstraction to demonstrate the point (neither the accuracies nor probability weightings are quite right for the more complicated trials used in the paper).

The explanation above and the code used to generate this figure are now included in the new supplementary materials (section 1.1). We updated the main text in the paper to read as follows, hopefully this is clearer:

The reason for this is that, while the average number of signal events remains constant across k, the distribution of the number of signal events, in a given trial, has some variability. This is explained in more detail in Supporting information 1.1.

Also, what do the parameters of the NLF look like for different values of trained k? The authors could provide a direct explanation of why the models fail to generalize when trained on k=1, based on these parameters. (comparing NLF to NLF2 should still be tractable for example)

As suggested, we have explored the parameters of LF, NLF, NLF2 and NLF3 trained on all values of k. We have added these figures, and a concise summary of these results to the supplement. In general, these models weight observations (features) with a bias in one direction (e.g. left in both channels for two time-steps) highly, and ambiguous observations (e.g. left in one channel, right in the other, for two time-steps) with weights close to zero. Which is as expected for our setup, in which each channel is equally reliable.

When trained on k=1, we do see that NLF2 and NLF3 learn distinct weights from when k>1. As when k=1, there is no temporal structure for the models to learn from. However, we have not quantified this difference.

Minor comments

It may be trivial but I think it would be useful to mention what a linear fusion algorithm that has a w timestep window would be/do? Would it be equivalent to the linear fusion algorithm without a window (because it accumulates over time)?

The two would be equivalent, as when summing information, the order of operations does not matter. For example, 10 + 5 + 5 = (10 + 5) + 5. We, agree that this would be useful to mention and have added it to the start of the "Incorporating time into multisensory algorithms" section.

Paragraph 3.2.1: "even when those sequences are longer than their window length (or memory)" – I couldn't get an intuition of why, it would be helpful if the authors could unpack it a bit?

To clarify this we have added the following: "*This is likely due to the fact that, even when k is high, short bursts are sufficiently informative of the prey's direction of motion; as they are unlikely to occur in the incorrect direction.*"

Figure 2: I am not sure I understand the correspondence between B1-D1 and B2-D2 -- e.g. NLF-3 trained on k=1, in C1 the max difference in accuracy is ~3, and in C2 it's 6. Aren't the dark blue lines supposed to be the same?

This difference is due to a normalization which we apply in panels B2-D2. We have added an explanation of this to the figure caption.

To allow a fairer comparison of parameters numbers, it could be interesting to test smaller sizes of RNNs, and see when the RNN's performance starts to break.

As suggested, we trained a set of smaller RNNs, with an equivalent number of parameters to NLF3. Interestingly, these smaller RNNs perform only as well as LF/NLF and worse than both NLF2 and NLF3 across most values of k (we have included a figure showing this in supporting information 1.2). This could suggest NLF_w to be a better model per parameter than the RNN. However, we would want to perform a larger hyperparameter search before concluding this definitively.

I find Figure 4 quite hard to read (e.g., what is gray?), and was wondering if 3 heatmaps (one per model) side by side with the same color scale wouldn't be easier?

We fully agree with this, and have updated the figure as suggested!

Reviewer #2:

The paper presents a model that accounts for multisensory integration and time. The models are tested using a novel task which is motivated by the example of hunting a prey. The paper presents five models, two with temporal independence (Linear Fusion, LF; and Non-Linear Fusion NLF), two which account for time (NLF two time steps, NL2 and three time steps, NLF3) and an RNN as a comparison. Overall I think the paper shows some very interesting findings. My comments are really focused on clarifications and further details about the models and the results.

Introduction

The introduction is concise and readable.

Two behavioural papers that do consider the trial by trial impact on behaviour that might be of interest are Wozny & Shams 2011, Shaw et al. 2020. This does raise an interesting debate about whether the benefit seen in a multisensory trial is partially due to a repetition of signal.

The use of the Lèvy distribution is interesting and could be mentioned in the intro to telegraph it being used later in the methods.

Thank you for these suggestions, we have added both references and a mention of the Lévy distribution to the introduction.

Results

I appreciate that the format of PLoS papers has the methods at the end but some more detail about the models in the results would be nice, like number of parameters is 3^{wNc} and the RNN uses ReLUs.

In line with this suggestion, we have added each model's parameter scaling function to Table 1 and some additional details on the RNN to the results section.

In Figure 2 that caption states accuracy and standard deviation of each model but the only model with visible error bars is the RNN, why is this?

Thank you for highlighting this! For the non-RNN models, we fit one model per value of k. For the RNNs we fit 5 models per value of k and so can report a distribution across models. We have clarified this in the figure caption.

Some more detail about the structure of the Lèvy distribution would be nice, just a sentence.

We have added an extra sentence on this, to the first paragraph of the "Capturing multi-timescale structure" section.

Are the results in Figure 3 train and tested on the same k? Also, in Figure 3 is the highlighted area trained on mixed distributions while the dashed lined are trained on only levy or only uniform. And is the highlighted area representing the standard deviation.

Sorry for the confusion. We have updated this caption to describe this figure more clearly!

Figure 4 would benefit from a bit more explanation in how to interpret the results. One possible way of helping the reader for example would be to box the lower right 4x4 grid for Test 5-8 and Train 5-8 where the RNN is "winning".

Thank you for the suggestion! Though in line with reviewer 1's recommendation, we have replaced this figure with an image per model – which should make the results easier to interpret.

Discussion

Like the introduction, the discussion is readable and concise.

Methods

While it is important to show that the equations can generalise to w time-steps and N_c channels. As the paper focuses on 2 and 3 time steps and two channels it might be nice to show an example of these simpler versions, only a suggestion.

We added an example for N_c=w=2 to the Methods section describing NLF_w.

For the simulations in the paper what values or values of the task parameters (such as pg and pe used)?

Thank you for noting this omission! We have added a section to the methods which include these values.

Just to a clarification could the burst be in one or both channels Xit and if so did the authors consider training a single channel version or a version with burst only on a single channel, to simulate a unisensory task.

During the burst (when the emission variable e=1), both channels have a higher likelihood of signaling the correct direction (p_c) than outside of the burst (when e=0). As such, bursts will be composed of some combination of uni and multisensory signals. Hopefully, the new panel (Figure 1G) makes this clearer.

We have now trained and tested a set of unisensory models on our current task (Figure 2A).

Altering the task itself, such that one channel bursts and one does not, would move us into scenarios where each channel carries distinct information. This would be great to explore, but seems rich enough for a future project!

5.2.1 should have the subheading Detection task with k timesteps (Detk)

Updated!

How long did it take to train the models?

On the hardware we used our LF and NLF models trained in < 30 mins. While NLF_w required around 8 hours, and our RNNs around 15. Though, for someone interested in running similar simulations, these times will vary with the task hyperparameters (e.g. the number of trials), the model properties (e.g. the size) and the hardware used.

The RNN could have its own subheading.

Agreed! We have split the inference section into NLF_w and RNN.

References

Wozny, D. R., & Shams, L. (2011). Recalibration of auditory space following milliseconds of cross-modal discrepancy. Journal of Neuroscience, 31(12), 4607-4612.

Shaw, L. H., Freedman, E. G., Crosse, M. J., Nicholas, E., Chen, A. M., Braiman, M. S., ... & Foxe, J. J. (2020). Operating in a multisensory context: Assessing the interplay between multisensory reaction time facilitation and inter-sensory task-switching effects. Neuroscience, 436, 122-135.
